# Mindfulness-based interventions: what more can the West learn from Buddhism? A fieldwork study

**DOI:** 10.3389/fpsyg.2025.1579575

**Published:** 2025-06-23

**Authors:** Andrew Boxer, Frances Shawyer, Ian Coghlan, Debbie Ling, Graham Meadows

**Affiliations:** ^1^Department of Psychiatry, Monash University, Clayton, VIC, Australia; ^2^Department of Social Work, Monash University, Clayton, VIC, Australia

**Keywords:** mindfulness, Buddhism, mindfulness-based intervention, MBI, meditation, qualitative research, psychology, Western world

## Abstract

**Objectives:**

Mindfulness has been adopted from its origins in Buddhist philosophy and is now widely applied in Western psychology. Existing research suggests this has been beneficial. The aim of this study is to identify possible areas for further integration of Buddhist approaches into current mindfulness-based interventions (MBIs) from the perspective of individuals from a range of geographical areas who have had exposure to mindfulness and traditional Buddhism.

**Methods:**

Participants were attendees of a 30-day Lam Rim meditation retreat. Data collection involved an online survey (*n* = 42) and subsequent follow-up semi-structured interviews (*n* = 11). Qualitative thematic analysis was used to explore the differences between traditional Buddhist and Western Psychological approaches to mindfulness and how Buddhist understandings might be further integrated into Western MBIs.

**Results:**

Participants noted that the Western definition of mindfulness remained unclear compared to specific Buddhist understandings of mindfulness. While Western applications of mindfulness often focused narrowly on health and productivity outcomes, traditional Buddhist perspectives offered a broader worldview encompassing a life philosophy. Some broader Buddhist concepts and practices identified by participants as potentially helpful to the West include impermanence, Buddhist mind science, the Four Noble Truths, emptiness, dependent arising, and compassion.

**Conclusion:**

Some of the identified concepts and practices such as compassion have already been developed into detailed secular frameworks, but further integration is possible. Future research could also continue secularizing deeper Buddhist perspectives, such as Buddhist mind science, to enrich mindfulness practices and promote holistic mental health and well-being while maintaining a non-religious framework.

## Brief history of mindfulness

Over the past four decades, mindfulness has gained increasing traction as an intervention in Western Psychology. This practice has been largely derived from Buddhist mindfulness, which emerged in India some 2,500 years ago (Husgafvel, 2016). By being and practicing mindfulness, individuals learn to notice and question the associations between stimuli/events and the automatic thoughts, feelings, and physical sensations that arise in response to them (Analayo, 2003; Dunne, 2015; Shapiro et al., 2006; Thanissaro and DeGraff, 2012). Becoming conscious of automatic processes provides an opportunity to interrupt the flow of problematic emotions and suspend habitual patterns of thought. Traditionally, this type of intervention may also be applied to gain insight into progressively deeper levels of mind and the reality accessed at these deeper levels.

Within the last century, mindfulness has gained attention in the West through the work of several key scholars and spiritual teachers who taught Buddhist practices to Western students. For example, Thich Nhat Hanh, a Vietnamese Zen master, opened 11 monasteries worldwide and had 700 monastic disciples and lay teachers when he died in 2022 (Dorbaire, 2023). Another notable figure was Lama Thubten Yeshe, a Mahayana Tibetan Buddhist practitioner, who created The Foundation for The Preservation of The Mahayana Tradition (FPMT) in 1975 and opened up several traditional Tibetan monasteries around the world (Yeshe, 1998). FPMT gained prominence by offering a one-month Lam Rim meditation course for Westerners every November since 1971 at the Kopan Monastery, Nepal, where the fieldwork for this paper was based. In the 70’s, Professor John Kabat-Zinn, developed a mindfulness-based stress reduction program (MBSR) (Kabat-Zinn, 2003). MBSR was one of the first programs to integrate traditional Buddhist practices with modern psychological techniques.

Since these developments, mindfulness has become increasingly popular in Western culture, with the development of various mindfulness-based interventions (MBIs) in psychology and healthcare (for review, see Zhang et al., 2021). MBIs are often used to treat a variety of mental health conditions, such as anxiety and depressive disorders, and to improve general well-being. These interventions may include guided meditations, mindful breathing exercises, other mindfulness-based practices, and incorporate elements from other therapeutic approaches, such as Cognitive Behavior Therapy [e.g., Mindfulness-Integrated Cognitive Behavior Therapy (MiCBT)] (Cayoun et al., 2018). Mindfulness has also expanded from psychology and health care and into other applications, such as organizational psychology (Zhang et al., 2021), for example, work-related stress, engagement, and job satisfaction (for review, see Kotera, 2021); correctional settings (for review, see Tadros and Urais, 2023); positive psychology, for example, enhancement of eudaimonia and hedonia, pro-social behavior, cognition and performance (for review, see Kotera, 2021); and education, for example, student well-being (Sapthiang et al., 2019).

Despite the growth of mindfulness in the West, significant differences remain between traditional Buddhist and Western psychological approaches. These differences stem from historical, cultural, and philosophical variations (Phan-Le et al., 2022). While both emphasize mindfulness for well-being, they can diverge in conceptualization and practice. Some scholars criticize the western interpretation and the separation from its Buddhist roots, yet even within Buddhism, different traditions interpret mindfulness in distinct ways (Dunne, 2015). As this study is set in a Tibetan monastery of the Gelugpa tradition, its exposition and discussion will primarily draw on related philosophical texts and treatises.

### Mindfulness in traditional Buddhist and Western psychological approaches

#### How traditional Buddhists utilize mindfulness

Mindfulness has several uses within the Buddhist framework, serving various purposes aimed at spiritual development and ethical living (Analayo, 2003; Thanissaro and DeGraff, 2012). It constitutes one of the stages within The Eightfold Path, a foundational set of eight Buddhist values or a code of conduct for liberation from suffering. Rooted in the Sanskrit term “smṛiti,” meaning “remembrance” or “the act of calling to mind,” mindfulness underscores the importance of constantly recalling and focusing on living a virtuous life while striving to detach oneself from worldly attachments (Analayo, 2003; Thanissaro and DeGraff, 2012). This practice enables Buddhists to embody moral teachings and pursue the ultimate goal of transcending the cyclic existence of life, death and rebirth. While contemporary interpretations may overlook the significance of these aspects, they are crucial in understanding the broader implications of mindfulness practice within the Buddhist traditions (Thanissaro and DeGraff, 2012).

According to Tibetan Buddhist texts on the nature of mind called “Lorig” (Tib. *blo rig*), mindfulness is one of 51 mental factors essential in Buddhism for understanding how the mind and consciousness work (Thanissaro and DeGraff, 2012). Buddhism uses meditation practices to strengthen the mental factors that are beneficial to the mind and mental health and lead a wholesome lifestyle in line with the teachings of the Buddha. The West is familiar with mindfulness meditation but in Buddhism, mindfulness meditation is used in tandem with other meditations, of which mindfulness is just one.

Within the framework of mental factors, mindfulness is classified as one of the five “object-determining mental factors.” The five object-determining mental factors consist of; aspiration, conviction, mindfulness, concentration and knowledge. These factors enhance focused attention on specific object characteristics, crucial for spiritual maturation and meditation practice (Feldmann, 2016). They are present in most moments of consciousness and can be virtuous or non-virtuous, depending on the object and other mental states associated with it.

The role of mindfulness in this framework is to recollect the object through three key mental functions; maintaining familiarity with the object, avoiding forgetfulness, and remaining undistracted (Feldmann, 2016). In other words, to be continually bringing the object back to one’s mind and holding it there. This process of continuous engagement cultivates single-pointed concentration. When this deep concentration is fully developed, the mind becomes absorbed in the object, leading to heightened awareness and mental clarity, an experience known as “samādhi.” This refined focus is said to enhance one’s ability to analyze problems with greater precision and insight, ultimately fostering greater wisdom.

Mindfulness here plays a crucial role in nurturing, enhancing, and conditioning wisdom. According to this view, by fostering mindfulness, individuals can access and apply wisdom more effectively, thereby cultivating wholesome actions, ethical conduct, and ultimately, embarking on the path to liberation. Additionally, mindfulness serves as a foundation for generating temporary antidotes to afflicted mental factors like anger or pride, offering a means to counter-act their detrimental effects. Ultimately, mindfulness contributes to the cultivation of wisdom, enabling individuals to dispel delusions and progress toward liberation (Analayo, 2003; Thanissaro and DeGraff, 2012).

In traditional Buddhist teachings, mindfulness holds a central place within its framework and is further delineated into the Four Foundations or Applications of Mindfulness. These ‘foundations’ encompass mindfulness of the body, feelings, mind and Dharma as objects of contemplation. Dharma in this context refers to observing mental phenomena through the lens of core Buddhist principles. While modern interpretations of mindfulness predominantly emphasize the body and feelings, there is a notable gap in mindfulness of the mind and Dharma (Analayo, 2003; Thanissaro and DeGraff, 2012). As further discussed below, mindfulness of one’s body sensations and feelings provide some space and re-evaluation of stimuli, but the mind and Dharma are foreign objects of contemplation in Western psychology or MBIs. While, some programs focus on the awareness of mental states, traditional Buddhist mindfulness goes beyond the mental focus present in some MBIs.

The Buddhist concept of the *mind*, a subtle form of energy that continues from one life to the next, and *Dharma*, the Buddhist view of phenomena, cosmology and individual existence, are fundamental parts of the Buddhist teachings. These foundations are central to the core beliefs of Buddhism, particularly the belief in reincarnation and Karma. Dharma is also often associated with Buddhist fundamental teaching known as the Four Noble Truths. Buddha’s first teachings were the Four Noble Truths, and foundational to Buddhist philosophy, explain the nature of suffering, its causes, its cessation, and the path leading to its cessation. Thus, traditional teachings highlight the interconnectedness of mindfulness with reincarnation and karma, underscoring the ultimate goal of liberation across the continuum of existence.

#### How the West utilizes mindfulness

There has been some criticism of the Western application of mindfulness throughout its evolution, including the disconnection from its Buddhist roots. Buddhist scholars such as Thupten Jingpa have questioned whether it is acceptable to extract techniques like mindfulness then claim that these extracted techniques capture the essence of Buddhist teachings (Shonin and Van Gordon, 2016). He questioned whether it is contradictory to assert that a practice is both inherently Buddhist and simultaneously secular. Other scholars have argued that MBIs are not trying to be Buddhist and offer a secular presentation (Husgafvel, 2016; Kabat-Zinn, 2003; Monteiro et al., 2015).

Additionally, criticisms relate to aspects such as its definition (Chiesa, 2013; Davidson and Kaszniak, 2015), qualities such as non-judgment (Davidson and Kaszniak, 2015), ethical considerations (Berry et al., 2020; Monteiro, 2020), and evaluations and measurements (Chiesa, 2013; Davidson and Kaszniak, 2015). However, with each successive wave of development, some efforts have been made in areas to address these criticisms. For instance, some researchers have refined the concept of non-judgment and integrating ethical frameworks into mindfulness interventions (Purser and Milillo, 2015).

In Western psychology, Mindfulness is an intervention typically used to keep one focused on the present moment, detached from what has or will happen, to improve one’s mental health and unproductive/negative thinking (Phan-Le et al., 2022; Zhang et al., 2021). There is a strong emphasis on avoiding bringing bias and judgments to situations. Its focus is often to manage unhelpful automatic reactivity and a way to regulate emotions or improve cognitive functioning (Purser and Milillo, 2015; Shapiro et al., 2006; Zhang et al., 2021). Interventions are typically used to alleviate specific symptoms or conditions, such as stress or anxiety, or to promote emotional resilience. These practices may involve focusing on present-moment experiences, developing non-judgmental awareness, and cultivating a sense of compassion for oneself and others. Western interventions also often incorporate other Western psychological approaches such as Cognitive Behavioral Therapy (CBT) or Behavior Activation (BA) Therapy.

The West has interpreted and applied mindfulness in different ways from its traditional Buddhist origins. In adapting mindfulness for a secular audience, Western approaches have intentionally removed its spiritual and philosophical foundations to fit therapeutic contexts. As a result, mindfulness is often defined more narrowly and most commonly through Jon Kabat-Zinn’s definition, which is “the awareness that emerges through paying attention on purpose, in the present moment, and non-judgmentally to the unfolding of experience moment by moment” (Kabat-Zinn, 2003, p. 145). However, over time, some concepts of mindfulness have emerged, embracing a more comprehensive definition that preserves the core principle of moment-to-moment awareness but underscores the importance of qualities such as friendliness, compassion, and kindness (Dawson et al., 2020). Despite these advancements, conceptual and practical limitations remain, suggesting the need for further exploration and development in the field (Lee et al., 2021; Monteiro, 2020).

### Aim of study

This study aims to identify the differences between Buddhist and Western approaches to mindfulness from the perspective of individuals across a range of regions who have had exposure to mindfulness and traditional Buddhism. Additionally, it seeks to identify possible areas for further integration of Buddhist approaches into current MBIs.

This study does not seek to evaluate or suggest that Western MBIs are trying to represent Buddhism. On the contrary, the success of MBIs in the West is could be due to their adaptation of mindfulness techniques rather than a direct replication of Buddhist teaching (Krägeloh, 2013). Instead, this study aims to explore whether research *should* begin incorporating more Buddhism concepts, given the positive impact of the elements already integrated.

## Method

### Design

This study employed a qualitative thematic analysis design. Thematic analysis is a qualitative method used to identify and interpret patterns or themes within data and was used to analyze the qualitative data for several reasons (Böge et al., 2020). Firstly, it is not only a method of technical exploration but a way of demonstrating social, political and cultural context. This is critical in a project involving traditional and contemporary comparisons. Secondly, thematic analysis allows for the data collection and analytical process to inform the themes of the research process. This is important because it allows for exploration of themes surrounding mindfulness and its integration in Western psychology, without the confinement of preconceived ideas. Lastly, it is a well-structured, process-driven method that allows for reviewing and redefining themes and drawing generalizable conclusions about traditional and modern uses of mindfulness.

### Study setting

The Kopan November course is a 30-day Lam Rim meditation course held in Kopan Monastery in Kathmandu, Nepal each year. This is a highly intensive course. Each 5:30 a.m. to 9:00 p.m. (15.5 h) day consisted of; morning prostrations, 3 h of meditations, 4 h of teachings and one and a half hours of discussion groups, split up among three meals, and free time for further study, discussion or contemplation. Silence is maintained from dinner to after lunch the following day, with fasting and one meal a day for the last third of the course (10-day Mahayana Precepts). The 2022 course was translated from English live into different languages, including Spanish, Portuguese, and Russian.

The Lam Rim (“Stages of the Path”) are a collection of different texts and teachings presented in a gradual progression to describe the stages in the path to liberation (Tuladhar and Adams, 2003). The teacher has a variety of texts to utilize depending on their preference and audience. The core-strength of the Lam Rim is that it presents key Tibetan Buddhist teachings into a structured, gradual practice (Lama, 2013). It has been described as practical ‘Buddhism for Dummies’ manual for the spiritual path that outlines the major concepts in Buddhism (Sopa, 2004). From a Western, psychological point of view, the Lam Rim teachings have been described as a compendium of cognitive behavioral strategies to transform self-view, view of others and view of the world for the benefit of one’s mental health (Taylor, 2014).

### Participants

Participants were recruited from the 2022 Kopan Monastery November Meditation Retreat as it provided an opportunity for a broad demographic who, due to the course, has exposure to traditional Buddhist philosophy and mindfulness. Eligibility criteria were at least 18 years old, participated in the 2022 Kopan Monastery November Meditation Retreat, and be proficient in English. All participants were provided with a statement about participation explaining the study’s purpose and instructions and were informed that they can withdraw (consequence free) at any time. This study involving human participants was reviewed and approved by Monash University Human Research Ethics Committee (HREC) on 23/04/23 (Project Number: 36733) and had institutional approval from Kopan Monastery which invited participants to apply. Consent was implied by completion of the survey and recorded verbal consent was obtained prior to the interview. The retreat teacher (Geshe) also participated in the study and because of this person’s particular expertise, role attribution has been made for the Geshe’s comments to aid interpretation of the research findings. Because the Geshe was publicly identifiable, specific consent and HREC permission was obtained to identify their role in this way.

### Sample size

Power analysis is not applicable due to the descriptive nature of the data. However, based on the number of participants at the 2022 November Course (around 200), and an estimated response rate of 30% (Baruch and Holtom 2008; Cunningham et al., 2015), we anticipated sample size for the survey of approximately *n* = 60 which we expected would enable access to a useful range of opinions.

The sample size for interviews was based on thematic saturation. Qualitative researchers estimate the saturation point where the majority (70% to 92%) of themes are identified emerge in the first 5 to 12 interviews (Given, 2015; Guest et al., 2006; Guest et al., 2020; Morgan, 2002) and new incoming data is adding “little or no new information to address the research question” (Guest et al., 2020, p. 2).

### Recruitment

Recruitment involved convenience sampling from the 2022 Kopan Monastery November Meditation Retreat participants. Following the retreat, prospective participants were contacted with an invitation via an email from Kopan Monastery on behalf of the research team. The email included an invitation to participate in the study via a hyperlink along with further information about the study (e.g., explanatory statement and participation rights). Participants were also recruited using a reminder post to a private Facebook group with the 2022 Kopan participants. This post had the same content as the email reminder. Following of completion of this survey, participants expressing interest in a follow-up interview (by Zoom) were contacted via an email that included an explanatory statement and an invitation to participate in an interview at a mutually agreed time. The Geshe (retreat teacher) was invited directly to participate in an interview. There were no incentives for participation.

### Measures

#### Online survey

The survey included 17 open and one closed question which covered basic demographics (age, sex, country of birth, main language, and education) and themes relating to participants’ level of experience with Buddhism, meditation, and experience at the retreat. See Appendix A for the survey questions.

#### Semi-structured interview

The interview comprised 17 to 22 semi-structured questions depending on MBI experience. Questions expanded on participants’ level of experience with Buddhism, mediation, and experience at the retreat. The interviews had a greater focus on participants’ views regarding integrating a Western and traditional understanding of mindfulness. It explored what participants found helpful from the course and how this differed from what they had learnt previously in the West. See Appendix B for the interview questions.

### Procedure

The online survey was self-administered by participants via computer or smartphone at a time and location of their choosing. The study was hosted on the password-protected online survey platform Qualtrics. At the end of the survey, participants were asked if they wished to be contacted to participate in follow-up interview. During the interviews, the interviewer prompted participants for further detail when necessary. All interviews were conducted and recorded via Zoom, then securely uploaded to LabArchives, an encrypted cloud-based storage platform, for analysis. The recordings were first transcribed automatically using Zoom’s audio transcription feature and then manually reviewed and edited for accuracy.

### Data analysis

Thematic analysis was conducted following Clarke and Braun’s (2017) six-phase framework to systematically identify and interpret patterns within the data. The first step involved becoming familiar with the data and transcripts, taking initial notes on recurring ideas. This process allowed for an in-depth understanding of participants’ perspectives on integrating Western and traditional Buddhist mindfulness practices. The second phase involved initial coding, where meaningful segments of text were systematically labeled to capture key insights. Common Buddhist concepts raised during the questionnaire and discussed in interviews were used to inform this process. Coding was flexible, allowing new themes to emerge as the data were further analyzed. For example, raw data were grouped into themes such as “cultural tensions,” which included concepts like karma and rebirth, and ‘Buddhist mind science’, which reflected participants’ descriptions of what they found most helpful in traditional teachings. Thirdly, codes were reviewed and potential themes were constructed from grouping some of the codes intro broader themes, reflecting patterns across responses. Fourthly, the themes were reviewed and refined so that each code appropriately reflected the theme. Then, the themes were named, defined and refined, when appropriate, ensuring they accurately captured participants’ experiences and perspectives. Lastly, the author related the themes back to the aim of the study and a structured narrative was formed, contextualizing findings within the broader research framework. This was refined where needed.

### Reflexive statement

It is important to consider the researcher’s effect on data analysis, as qualitative research is inseparable from its context. In this study, the first author, AB, conducted the interviews and analysis for a PhD thesis. AB is an experienced psychologist in his fifth year of practice with early exposure to mindfulness through Tibetan Buddhism. He has studied with many teachers through FPMT, including Ven. Robina Courtin for 20 years and, more recently, Geshe Tenzin Namdak. He has completed the one-month meditation course at Kopan Monastery three times, participated in additional retreats and is currently in the final year of the FPMT five-year Basic Program. While his exposure to the Western use of mindfulness was limited before his PhD, he has since completed a certificate in MiCBT and gained extensive applied experience in the field.

AB’s background and the inherent subjectivity in qualitative research led to some assumptions about the study. He assumed that traditional mindfulness would positively impact individuals’ lives and that the West would oversimplify and modify its use to fit a reductionist model. Despite potential biases, AB’s experience provided useful insights during the interviews. His background assisted him in engaging with interviewees and asking questions about the core theoretical underpinnings of traditional and Western mindfulness.

## Results

### Survey results

The survey took 9 min and 35 s minutes to complete on average.

#### Participants

In total, 42 participants completed the survey.

The age range of participants varied from the 20’s to the 60’s and above, with most in their 30’s or 40’s. Almost two thirds were women. The participants were from 22 different countries, predominantly within Europe. At home, most participants spoke either English (33.3%) or Spanish (26.2%). This was a well-educated group, with 50% of the participants having completed a postgraduate degree and another third having completed a bachelor’s degree (see Table 1).

**Table 1:** Demographic characteristics of the survey sample.

Characteristic	% (*n*)
Gender	
Male	35.7% (15)
Female	61.9% (26)
Prefer not to say	2.4% (1)
Age group	
20’s	9.5% (4)
30’s	33.3% (14)
40’s	26.2% (11)
50’s	14.3% (6)
60’s	16.7% (7)
Country of birth	
Europe	52.4% (22)
South America	14.3% (6)
North America	11.9% (5)
Australia	9.5% (4)
India	7.1% (3)
Asia	4.8% (2)
Main language at home	
English	33.3% (14)
Spanish	26.2% (11)
German	7.1% (3)
Portuguese	7.1% (3)
Other	26.2% (11)
Education	
Secondary school	7.1% (3)
Diploma (inc. associate diploma)	7.1% (3)
Technical qualification	2.4% (1)
Bachelor’s degree	33.3% (14)
Postgraduate qualification(s)	50.0% (21)

Almost a third of the participants (30%) had completed a Western MBI (*n* = 9). Participants had a range of spiritual backgrounds but over 80% considered themselves Buddhist currently (see Table 2). Almost half (47.6%) of participants had over 10 years active interest in Buddhism, with another 23.8% having 5 to 9 years of active interest. Over 75% of participants had spent more than 5 days in a retreat and 54.8% reported that they meditate at least once a day. The three meditations practiced most often by participants where: analytical (40.5%), concentration/shamatha (38.1%) and mindfulness (19.1%) and 17.1% reported that they had previously completed a mindfulness-based Western intervention (e.g., MBSR).

Participants had a range of spiritual backgrounds but over 80% considered themselves Buddhist currently (see Table 2). Almost half (47.6%) of participants had over 10 years active interest in Buddhism, with another 23.8% having 5 to 9 years of active interest. Over 75% of participants had spent more than 5 days in a retreat and 54.8% reported that they meditate at least once a day. The three meditations practiced most often by participants where: analytical (40.5%), concentration/shamatha (38.1%) and mindfulness (19.1%) and 17.1% reported that they had previously completed a mindfulness-based Western intervention (e.g., MBSR).

**Table 2:** Spiritual/religious characteristics.

Characteristic	% (*n*)
Spiritual background	
Buddhist	39.7% (23)
Spiritual	20.7% (12)
Christian	13.8% (8)
Non-religious	15.5% (9)
Judaism	1.7% (1)
Hinduism	1.7% (1)
Other	6.9% (4)
Years interested in Buddhism	
<1 year	11.9% (5)
2	9.5% (4)
3	4.8% (2)
4	2.4% (1)
5+	23.8% (10)
10+	47.6% (20)
Days in retreat	
0–5 days	26.2% (11)
6–60 days	31% (13)
61–120 days	14.3% (6)
<120 days	21.4% (9)
Unsure	7.1% (3)
Frequency of meditation	
Multiple times a day	14.3% (6)
Daily	40.5% (17)
Multiple times a week	16.7% (7)
Weekly	11.9% (5)
Monthly	2.4% (1)
Only at retreats	7.1% (3)
Never	7.1% (3)
Completed a Western MBI	
Yes	30% (9)
No	60% (21)

#### Personal positive impact of Buddhism

Thirty participants responded to item 13 (see Appendix A) that asked what Buddhist-based concepts had the most positive impact on their lives. The top responses were compassion (*n* = 11; 37%), impermanence (*n* = 10; 33%), Death (*n* = 8; 27%), Karma (*n* = 7; 23%) and emptiness (*n* = 7; 23%).

#### Buddhist concepts considered valuable to add to Western MBIs

Table 3 shows the Buddhist concepts that participants felt would be valuable to add to Western MBIs. The most strongly endorsed were dependent arising (*n = 21*; 50%), attachment and aversion (*n = 21*; 50%), bodhicitta (*n = 20*; 47.6%), emptiness (*n = 17*; 40.5%), The Four Noble Truths (*n = 17*; 40.5%), karma (*n = 16*; 38.1%). The least endorsed was the hell realms (*n = 1*; 2.4%).

**Table 3:** Buddhist concepts endorsed as valuable to add to Western MBIs.

Buddhist concept	Valuable additions to Western MBIs *%(n = 42)*	Concepts in tension with pre-existing culture *%(n = 23)*	Most positive impact *%(n = 30)*
Dependent arising^*^	50.0% (21)	4% (1)	14% (4)
Attachment and aversion	50.0% (21)	-	-
Bodhicitta^*^	47.6% (20)	13% (3)	37% (11)
Emptiness^*^	40.5% (17)	9% (2)	23% (7)
Four Noble Truths	40.5% (17)	-	-
Karma^*^	38.1% (16)	35% (8)	23% (7)
Rebirth	14.3% (6)	35% (8)	10% (3)
Boddhisatva^*^	11.9% (5)	-	-
The Hell Realms	2.4% (1)	13% (3)	-

^*^A brief explanation of some Buddhist terms: Dependent arising—The Buddhist principle that all phenomena arise in dependence upon multiple causes and conditions, and nothing exists independently or in isolation. Bodhicitta—The compassionate aspiration for enlightenment for the benefit of all sentient beings. Emptiness—the understanding that all phenomena lack inherent, independent existence. Karma—The law of cause and effect where intentional actions create future consequences. Boddhisatva—This is a being who vows to delay their own liberation in order to help others achieve awakening.

#### Buddhist additions to Western MBIs suggested by participants

##### Possible improvements to Western meditation

A total of 29 participants (55.8% of the sample) responded to item 17 (see Appendix A). Within this group, seven participants (16.7%) expressed the view that the West could derive advantages from a more precise definition of meditation, delineating what it entails and what it does not. They observed a lack of clear purpose for meditation in the Western context, especially when contrasted with the more distinct purposes often associated with traditional Buddhist practices. Further insights into this perspective are discussed in the subsequent section, “Traditional Definitions of Mindfulness,” where interview results are explored.

Participants had a particularly negative view of how meditation is practiced in the West. One participant wrote that meditation is not ‘*woo-woo stuff’ (Participant s8), ‘navel-gazing’ (Participant s15)* or *‘a tool to make yourself comfortable or blissed out’* (Participant s4). Participant s8 furthered their comments by writing:

*Meditation is not about emptying your mind of thought. Meditation is not about chilling out and relaxing. Meditation is a very conscious, directed way of focusing the mind, in order to benefit self and others.*

Participants commented on the variety of meditations available in Buddhism compared to the West, in particular, the use of analytical meditation. There are several types of meditations in Buddhism beyond the already popular mindfulness and present awareness meditations. Buddhism has lesser-known meditations that require concentration and analysis of problems, as well as meditations that involves complex visualizations (e.g., Tong Len). Participants felt that the West could benefit from these more uncommon meditations.

*The East or Buddhism has much more experience and many more techniques for different user profiles.* (Participant s10)

##### Possible improvements to Western mindfulness

A reoccurring theme, but something that has already been incorporated to a greater or lesser extent into Western MBIs, gathered from survey question 16 for example (see Appendix A), was the inclusion of compassion and its impact on others. Mindfulness helped participants re-evaluate their thoughts, actions and the impact on other people. Participants mentioned incorporating a compassionate or altruistic motivation to engage in mindfulness could be greater than the motivation to benefit oneself. For example, participants felt that the focus should include the benefits to others through reflection on one’s own actions and how they affect others (negatively or positively).

*Mindfulness is not about chewing a raisin for twenty minutes. Mindfulness certainly translates to existing in our ‘normal day world’ in a more connected way; but it’s beyond this, connecting us to ‘others’ in a more compassionate way. Mindfulness is about helping others, helping ourselves, and creating a more compassionate world.* (Participant s13)

#### Buddhist additions to Western MBIs suggested by past MBI participants

Almost a third of the participants (30%) had completed a Western MBI (*n* = 9) according to survey item 15 (see Appendix A). These participants felt that generally Western MBIs failed to capture the essence of its Buddhist origins and could benefit from the more traditional Buddhist concepts of impermanence, ‘Buddhist mind science’ (a Buddhist view of how the mind works), emotions and perception, the Four Noble Truths, emptiness, dependent arising and compassion.

*Western mindfulness programs I have completed seem shallow compared to Buddhist courses/retreats I have completed. It’s as though these Western practitioners have extracted “bits” of the practice that suit the product they’ve created…rather than creating something that delves into the detail and essence of a genuine Buddhist practice.* (Participant s5)

#### Concepts or practices that are in tension with culture/tradition

Twenty-three participants responded to the question regarding the most challenging Buddhist-based concepts or practices that were in tension with their pre-existing culture. The most prevalent challenge, identified by eight participants (35%), pertained to the concepts of Karma and Rebirth. This was followed by four participants (17%) who expressed challenges or tension with guru devotion, mysticism/spiritual aspects. Interestingly, four participants (17%) also reported finding ‘nothing challenging or in tension.’ Additionally, two participants (9%) found aspects such as meditation, emptiness, and the hell realms to be challenging. These responses represent instances where at least two participants shared common perspectives in addressing the open-ended question.

### Interview results

The interview took on average 52.12 min to complete. The study conducted 11 interviews in which data saturation was achieved. All participants answered all questions.

#### Participants

In total, 11 participants participated in the interview.

Table 4 shows the demographic characteristics of the participants who were interviewed. The age range of participants varied from the 30’s to the 60’s and above, with the majority in their 30’s (55%). The participants were from eight different countries and over two thirds were men. Their personal background was most frequently Buddhist (55%), but participants often came from a Christian family background (64%). Mirroring the survey results, the interviewees were well-educated, with 55% of the participants having completed a postgraduate degree.

**Table 4:** Demographic characteristics.

Characteristic	% (*n*)
Gender	
Male	73% (8)
Female	27% (3)
Age group	
30’s	55% (6)
40’s	18% (2)
50’s	18% (2)
60’s	9% (1)
Country of birth	
Europe	55% (6)
Australia	27% (3)
Other (UK and NZ)	18% (2)
Education	
Secondary school	9% (1)
Bachelor’s degree	36% (4)
Postgraduate qualification(s)	55% (6)
Background (personal)	
Christian	9% (1)
Spiritual	18% (2)
Non-religious	18% (2)
Buddhist	55% (6)
Background (family)	
Christian	64% (8)
Spiritual	9% (1)
Non-religious	9% (1)
Hindu	9% (1)

### Overview

Ten participants and the teacher completed interviews with 34 themes/items identified. Between 1 and 11 interviewees made references to each item and there were between 1 and 32 references made to each item. All 11 interviewees mentioned religious differences, the definition of mindfulness, and the hardest parts of Buddhism. The most referenced themes/items were; the difference between the West and Buddhism’s use of mindfulness, the definition of mindfulness, and possible Buddhist suggestions to Western MBIs.

### Diversity and ambiguity of Western conceptualizations of mindfulness

As noted earlier, various definitions and applications of mindfulness have been proposed by mindfulness scholars (Choi et al., 2021; Lundh, 2020; Shapiro and Weisbaum, 2020). Such diversity was particularly evident in the answers related to the subheading *Mindfulness-related questions* (see Appendix B), where participants commented that.

‘*there are so many types of mindfulness you can find everywhere’* (Participant i5). One participant discussed her exposure to mindfulness in a corporate setting and how, in this context, mindfulness was used to increase productivity:

*We can do mindfulness and get your productivity up…It’s like we’re going to calm you down and get you focused and you’re going to be more productive*. (Participant i6)

Others commented on mindfulness ambiguity and that its often used synonymously with meditation or this general undefined sense that often “*connects mindfulness more with Western spirituality”* (Participant i5). Often, mindfulness was described or reflected on as a tool, vehicle or technique associated with awareness *“that requires your focus and brings you into the present moment”* (Participant i9). Like the popular definition by Kabat-Zinn mentioned above, comments reflected notions of non-judgmental present awareness or paying attention from moment to moment [Kabat-Zinn, 2003, e.g., *being in the present, non-judgemental* (Participant i9)]; *being in the present moment quote unquote* (Participant i1); *This kind of feeling, of deep awareness. or abiding in very* var*ious aspects of what’s happening at this moment, like in this moment* (Participant 2).

#### Buddhist definitions of mindfulness

Some participants emphasized the importance of remembering in mindfulness, both in terms of the practice of meditation (remembering to return to the breath) and in terms of remembering one’s Buddhist intentions. This emphasis was particularly evident in the answers related to the subheading *Mindfulness-related questions* (see Appendix B):

*Why do you train in this mindfulness [breathing Meditation]? Because when you try to count your breath from one to 10 and start over, counting as soon as a thought comes up. But alertness should be aware of that. And with mindfulness, you should be remembering what you’re supposed to be doing. And that’s the counting of the breath. And so then, whenever there’s a distraction, or you hear something outside? You be aware of it with alertness and with mindfulness, you say? Oh, no! I’m supposed to be doing the counting on my breath.* (Geshe)

Here the Geshe linked concentration forms of meditation that involve primary focusing on the breath with analytical forms. In concentration practices, practitioners concentrate solely on a sensation of the body (e.g., the breath entering and leaving the body) but can also include holding visualizations in the mind, particularly in Tibetan Buddhism (Guillaume et al., 2020; Shaw, 2021). In contrast, analytical practices involve the active engagement of the mind in contemplation and analysis of specific topics or concepts. This may involve contemplating philosophical questions, guided visualizations or reflections. Unlike concentration meditation, analytical meditation encourages cognitive exploration and reflection on various aspects of the chosen subject.

Participants often perceived both concentration forms of meditation, like breathing meditations, and analytical forms, like guided meditations, as interconnected or complementary practices within the broader spectrum of meditation techniques. Expanding on this, the participant goes on to say that a Buddhist practitioner is encouraged to not just be mindful of one’s thoughts but judge and question whether the thoughts/actions are beneficial:

*…then you come to the conclusion that this destructive emotion ‘hatred’ does not really bring any benefit to anyone right? So, you come to that conclusion, and the more you reason that the more you think about it, it produces an insight into your mind right? And these insights you can remember with mindfulness. So that’s kind of in that way mindfulness goes a step further than just being a bare awareness or just being ‘non taught’, or try to just relax right? So this actually tackles the problem or actually transforms our problems. Right? So that’s kind of yeah. And it’s it needs a bit more homework, I have to admit. But on the long term. It brings more benefit. Right?* (Geshe)

#### Differences between Western and Buddhist definitions of mindfulness

Participants commonly distinguished between their conceptualizations of what mindfulness encompasses from a Western perspective and the traditional Buddhist viewpoint. Some participants considered that the Western viewpoint was defined less clearly compared to the traditional Buddhist perspective, even though there are still differences among lineages (Dunne, 2015). Such differences were particularly noted in the answers relating to the subheading *Integrating the West and Buddhism* questions (see Appendix B).

*It’s [mindfulness’] kind of like became really vogue and popular, and it’s like everything’s mindfulness, and that maybe confuse me initially. because that’s probably where I first heard the word and then the Kopan’s [monastery’s] version of mindfulness is remembering, which is also, I think, an interesting take on mindfulness.* (Participant i7)

Participants described the Buddhist definition of mindfulness as being different to the Western approach, and its conceptualization and application also differed.

*When it’s [mindfulness] practice in the Buddhist context. Usually, it’s with a view of trying to achieve something specific or trying to, you know, there’s a theoretical framework behind it that there’s a reason why you’re practicing mindfulness rather than just what seems often in the Western context, that is kind of just these are tools that anybody could use. That will help you in some way or the background of, you know, you’re trying to eliminate suffering and be aware of how your mind works ultimately not just become somehow more aware, and that will just be helpful for you in some way often to be more productive. Or what have you? Which is very different from the Buddhist orientation.* (Participant i1)

Complementing the survey responses, interviewees also remarked that the Buddhist version/definition of mindfulness was more expansive. Comments suggested the Western definition was more specific in its scope targeting one’s health, productivity or stress outcomes. Comments associated with traditional Buddhism involved a broader definition relating to a world view encompassing a way of life and provided participants with something more than improving health outcomes.

### What participants found helpful about mindfulness from a Buddhist perspective

Complementing the results from the survey data, some participants suggestions have already been integrated into the west which they might not have been aware of. Such suggestions were particularly noted in the answers relating to the subheading *Integrating the West and Buddhism* questions (see Appendix B). For example, some participants commented on how helpful learning about compassion was and suggested incorporating it into mindfulness practices.

*I think the sort of like deeper teachings they have on compassion is really helpful. So, I think we know that there’s a word ‘compassion’ in our societies but I don’t think we really understand it is true meaning, you know. I think we try, we would see compassion, as is empathy. We see it is like looking at somebody like in the street who’s homeless having a really tough time, and we’d be like that’s really bad, you know. That’s really tough, you know, when we get it we feel something like we all often feel quite awkward, right. But the actual definition [of compassion] for Buddhism is like to see the suffering and to take action. You know it’s to relieve the suffering. So, I think that kind of aspects for yourself and for other people is really useful.* (Participant i2)

Other suggestions already part of Western psychology included concepts that are used in CBT. They emphasized how thoughts and feelings impact choices, and how reactions to stimuli are malleable. This parallels Buddhist teachings on attachment and aversion, which influence suffering through biases in perception. This is considered further in the discussion section.

### What participants found helpful from Buddhism that might benefit the West

#### Buddhism as a worldview

Overall, participants conveyed that they found the content from Buddhism helpful and that there were many insightful concepts beyond mindfulness. Such suggestions were particularly noted in the answers related to the subheading *Integrating the West and Buddhism* questions (see Appendix B). For instance, there were several comments regarding Buddhism as a worldview and a spiritual path:

*So to say that it’s more than just reducing thoughts. It’s not only about that, but it’s also about changing the way you interact with the world with yourself and learning to understand what your own mind is doing. You know what afflictions are and what the nature of reality is. So, this is all things that will not come just from doing mindfulness meditation.* (Participant i3)

Participants also found that a lot of Buddhist concepts and principles were or could be effectively presented in a secular way and that some of the more spiritual aspects, that were hard for them to assimilate, could potentially be separated from the broader teachings.

*In Buddhism we also have secular aspects. Yeah, like philosophy and psychology, for example. So those aspects of mind science or psychology and philosophy, they can be practiced and studied by the non-believer. Whatever you believe in right, it can be done in a secular, universal way.* (Geshe)

#### Buddhist mind science

Additionally, participants mentioned, in the interviews related to the subheading *Integrating the West and Buddhism* questions (see Appendix B), the concept of a Buddhist mind science. Buddhist scholars have developed detailed framework for the phenomenon of the mind and consciousness. This consists of 51 mental factors (qualities of the mind) with an explanation on how each of these factors interact with mind and consciousness.

*The Buddhist mind science and its philosophy, so to say is the secular or universal aspects of the mind and its philosophy… because it’s also important to know how things appear to the mind, the philosophy behind it, you know, because we all think when we see with anxiety or fear or depression, things appear to the mind, but it’s not a reality. It’s just a mental, creative reality by the individual, right?* (Geshe)

Several participants remarked on how the Buddhist explanation of the mind, thoughts, and consciousness, and their interaction with phenomena, greatly contributed to their understanding of mental health. They expressed that the Buddhist perspective facilitated a deeper comprehension of both themselves and the world, significantly impacting their overall well-being.

It is ridiculous, that we don’t already know just how our minds work, how consciousness exists for us from the first-person perspective. How do you think I’d never really understood, like, what is a thought. And where is a thought in reference to a sensation? And then where does my visual field exist? (Participant i7)

#### Dependent arising and emptiness

Complementing what participants mentioned in the survey, in the subheading *Integrating the West and Buddhism* questions (see Appendix B), dependent arising and emptiness were also concepts that participants felt were important for the West to be exposed to [e.g. [gave me a] *better understanding of reality (Participant s3); Has a logical basis and is easy for western minds to understand (Participant s3)*]. These concepts are further explained in the discussion under the subheading *Dependent Arising, Emptiness and Interconnectedness.* In brief, dependent arising refers to the principle that all phenomena arise in dependence on causes and conditions, while emptiness refers to the absence of inherent, independent existence within those phenomena. Both concepts highlight the fundamental interconnectedness of all experiences and events.

*I think the hardest part is emptiness…In the developed Western world. We are very focused on the individual, and we perceive ourselves as individuals, a separate from the rest of the world from other people or from nature, and so on. And emptiness is basically about dissolving the ego and realizing that we are not separate…But we’re just a collection of stuff, and if you break it down into the individual parts you’re not going to find anything. And I think that’s a pretty shocking thing that you at first, when you come in contact with it, you think it’s complete nonsense. The more the time you spend on it, the more you analyze the more you realize that there might be something to it. Because there is nothing to be attached to? If you realize that everything is empty? Then what’s the point? I’m not attached to air. So if, as an analogy, if myself, my daughters, my car, my parents, if they’re all empty and just conceptualizing, that is here, why should I get attached to them? So why am I spending all this energy on holding on to something that doesn’t exist at all from its own side.* (Participant i4)

And

*I think dependent arising is pretty special…so anything that could be assembled by the mind can be deconstructed by the mind. So if you start to, if you have a lot of anxiety in life in general to know that your mind is one element you know, to know that your conditions, your living is elements coming together. Once you start to see that it’s not this solid, fixed thing that can’t be moved. And you start to see that, like if I play around with some of the elements, you know, if I change something in my external environment, if I change it in my internal environment. You start to see that you can like, Take the pieces apart a little bit. I think that, like more fluid approach on life is useful* (Participant i2).

## Discussion

This qualitative study explores the utilization of mindfulness in Western psychology compared to its traditional Buddhist context, through surveys and interviews with individuals from diverse geographical regions who have had exposure to mindfulness and traditional Buddhism. Findings indicate that while Western psychology has incorporated some beneficial Buddhist mindfulness practices, participants identified additional techniques from Buddhism that remain underutilized.

### Already incorporated suggestions

Participants recognized some commonalities between Western and Buddhist mindfulness practices, highlighting several beneficial elements from Buddhist mindfulness that have already been included into Western mindfulness practices. These elements encompass compassion therapy, the ABC (antecedents, behavior, consequences) model of CBT, and the acknowledgment of personal biases.

#### Compassion therapy

Compassion was a common suggestion, but whether participants knew of its role or other similar meditations (e.g., Loving Kindness) in Western psychology was unclear. Some comments indicated an awareness of compassion’s use in the West but suggested that integrating the Buddhist’s utility of compassion would be valuable. Given the strong evidence for compassion, often more powerful effects than mindfulness (Feliu-Soler et al., 2017; Kelly and Carter, 2015), there might be a case to be made that it should always form an explicit part of mindfulness training. At present, MBSR and MBCT (Mindfulness Based Cognitive therapy) does not include compassion explicitly, while MiCBT and does. Some MBIs, such as Mindfulness-Based Relapse Prevention (MBRP), integrate compassion more implicitly through self-compassion practices, which aim to reduce shame and foster a nonjudgmental, compassionate approach toward oneself and one’s experiences (Marlatt and Witkiewitz, 2005).

In Buddhism, compassion is incorporating a wish for people to be free of suffering. But the true meaning goes deeper and is incorporated in the Mahayana’s concept of Bodhicitta. Bodhicitta is the desire of becoming enlightened with the aspiration to help others and free them from suffering. This is close to the definition and concept used in Compassion Focused Therapy (Gilbert, 2009).

#### CBT

Other suggestions already part of Western psychology included concepts that are used in CBT. Some comments from participants aligned with the ABC (i.e., Antecedent/Beliefs/Consequence) function analysis model of CBT and how our thoughts/feelings can affect our choices, and how our reactions to stimuli are changeable. The Buddhist model parallels the ABC model through attachment and aversions and being more mindful of their impact on our suffering (David et al., 2014; Diaz and Murguia, 2015). Both believe in ‘A’ the activating event or a neutral stimulus. This is then influenced by our ‘B’ beliefs, or what Buddhist could refer to as our delusions that lead to our attachments (things we like and want more of) and aversions (things we do not like and want to decrease). These beliefs, delusions or attachments/aversions bias our views on the stimuli and lead to positive or negative emotions (Feldmann, 2016). Buddhists might refer to these biases as delusions that are imbued from the mind and are not accurate representations of reality (i.e., a lack of understanding of the Buddhist concept of emptiness). Clinging to a biased outlook (that something is more positive or negative than it is) is the cause of suffering or the consequence ‘C’. Although this is common in some MBIs such as MBSR and MBRP, this is not always explicit or presented in a similar way to Buddhism.

Exploring whether this alternative perspective could enhance CBT or facilitate its integration into other MBIs, such as mindfulness practice, presents an area for future investigation. Such integration is illustrated in MiCBT, which merges mindfulness and CBT through a co-emergence model (Frances et al., 2020). This model emphasizes mindfulness of bodily sensations during states of arousal and aims to cultivate an Equanimeous response to these sensations to mitigate reactivity.

The examples above illustrate the potential synergy between Buddhist theory, mindfulness, and some Western interventions. While elements such as compassion and certain cognitive-behavioral concepts like the ABC model have been integrated into Western psychological practices to some extent, there remains potential for further integration of Buddhist concepts. For example, the Buddhist concept of Bodhicitta or Tong Len offers strategies and insights into compassion that could deepen and extend existing therapeutic outcomes. Similarly, the understanding of attachment and aversion from a Buddhist perspective could provide a simple yet nuanced understanding of how beliefs and biases influence emotional reactions and psychological well-being. The potential to refine and enhance existing therapeutic approaches with a diverse, well-structured worldview framework that Buddhism offers could lead to more comprehensive, nuanced, and alternative interventions that better address the diverse needs of mental health consumers. Some suggestions on how to incorporate theses are further discussed below.

### Suggested underutilized Buddhist principles

#### Mindfulness’ definitions and boundaries

Over the last half-century, there has been an increase exposure to Buddhist concepts in Western societies, in particular, mindfulness. This cultural acceptance has led to positive scientific scrutiny, specifically through the application of mindfulness in the ‘70s, and an interest in Eastern meditation in general (Shapiro and Weisbaum, 2020). This cultural shift has created a receptive environment for exploring the more profound philosophical and contemplative dimensions of Buddhism within mindfulness, which strongly aligns with contemporary concerns related to mental health, self-awareness, and overall life satisfaction (Kang and Whittingham, 2010; Rogge et al., 2022; Van Gordon et al., 2015). The comments from participants seem to mirror this interest in more complex and nuanced Buddhist theories.

Participants identified distinctions in terms of definition and scope. Western mindfulness’ definition was viewed as less precise with a more limited application, primarily focusing on mental health outcomes, well-being and enhancing productivity. In contrast, traditional Buddhist mindfulness was regarded as having a well-defined structure, often integrating various facets of the mind into its practice and instruction. Moreover, it was regarded as an integral component of a broader philosophical and worldview that participants found helpful. Participants emphasized the expansive worldview inherent in Buddhism, which was seen as lacking in the narrower focus of Western mindfulness.

For example, participants reported benefitting from Buddhist mind science and the Buddhist perspectives on consciousness and perception. This is further expanded in the section *Buddhist mind science* below. While some Western approaches implicitly incorporate some of these perspectives on cognition, the absence of a similarly structured and precise framework reflects a distinction from the more well-defined conceptualization found within Buddhism. Participants also found other broader concepts from traditional Buddhism, such as emptiness and dependent arising, particularly useful in challenging their attachments to their ego and viewpoint, allowing them to perceive things from alternative perspectives. This practice is complex and requires nuanced understanding that can take years to develop. It is further discussed in *dependent arising, emptiness and interconnectedness* subheading below. These traditional elements were deemed helpful in their mindfulness practices. The broader perspective in Buddhism was seen by participants as valuable not only for addressing mental health concerns but also for gaining insights into the human experience.

Conversely, some of the less contemporary aspects, such as the spiritual aspects of Buddhism, are still difficult for Westerners to integrate/understand. Interestingly, some of the more challenging aspects were also reported to be the most beneficial. For example, although, a third of participants (35%, *n* = 8) reported that Karma was in tension with their pre-existing culture and traditions, a similar percentage of participants also felt karma would add value to MBI’s (38.1%, *n* = 16). These aspects, including whether their benefit is quantifiable and whether there are ways to make them more palatable to Western culture, are areas for future research.

#### The separation from traditional and spiritual Buddhist teachings

Navigating Buddhism’s mystic and spiritual components was the most challenging aspect for many participants. According to cultural adaptation theory, this tension is to be expected, as the more mystical or metaphysical aspects of Buddhism, such as karma and rebirth, are often not congruent with the positivist paradigm that underpins much of Western culture (Kim, 2017). However, precedents exist that distinguish the spiritual elements from the secular facets while preserving the authenticity and tradition of the teachings. For example, His Holiness the Dalai Lama (the spiritual leader for Tibetan Buddhism) has created secular ethics that aims to harmonize traditionalism with secularism (Irawan, 2018). This incorporates Buddhist ethics, doctrines, practices, and soteriology with contemporary ideals, avoiding the more traditional or spiritual practices found in Buddhism (Irawan, 2018). Stephen Batchelor is another example of someone who has done extensive work in separating secular aspects of Buddhism. For example, in his book “Buddhism Without Beliefs,” (Batchelor, 1998) he explores Buddhist philosophy and practices stripped of religious and metaphysical elements, making them more accessible to secular audiences. These examples demonstrate the ability to present Buddhism in a way that resonates with individuals interested in its practical benefits without the need for religious adherence.

This separation from traditional and spiritual Buddhist teachings was mirrored by the retreat teacher/Geshe, who described that Buddhism could be delineated into three categories: Buddhist mind science (or psychology), Buddhist ethics, and Buddhist spiritualism. As explored previously, the first two categories can be presented secularly and could be seen as taking the Buddhism, out of Buddhism.

Future research should investigate how to present these aspects of Buddhism in a secular way and their potential utility in Western psychological interventions. This could include MBIs but also extend to other areas, like His Holiness the Dalai Lama’s ethics. Such research could help consumers engage with Buddhist wisdom in a manner that aligns with their social and cultural preferences.

#### Is harder, better?

Buddhism involves a more extensive and intricate journey compared to some contemporary mindfulness techniques. While the path may be longer and more challenging, it claims to offer the potential for a more profound and holistic transformation. Buddhism is not just a set of techniques; it encompasses a comprehensive way of life and a broader worldview. This helpful comprehensive worldview was something participants noted.

Engaging with Buddhism can help individuals develop a more encompassing perspective on existence and their place in the world. It goes beyond addressing immediate concerns and delves into fundamental questions of purpose and meaning. This is akin to some of the principles of Acceptance and Commitment Therapy (ACT), which also emphasizes values and a holistic approach to well-being (Hayes, 2002). One key difference is that ACT encourages participants to create their own values, while Buddhism assigns them (Fung, 2015). Whether this assignment of values could be beneficial or a possible adjunct to ACT is something for future research.

ACT and Buddhism encourage individuals to consider their actions and choices within their broader values and life goals. Buddhism’s comprehensive values and perspective could lead to more enduring and meaningful changes in one’s life, complementing contemporary mindfulness practices that primarily focus on techniques and immediate stress reduction.

Additionally, as the number of mindfulness and other Buddhist derived interventions (BDIs) increases, so could the complexity of secular Buddhist theories, as stated above. Future research should investigate the utility of more complex holistic BDIs, exploring their potential to enhance therapeutic outcomes and mental well-being.

Despite this, it is important to acknowledge that MBIs are deliberately time-limited (i.e., 6 to 12 weeks), pragmatic interventions to target specific psychological symptoms. This focused approach is not necessarily a shortcoming but a design choice intended to maximize accessibility and clinical effectiveness that allows space for participants to explore broader spiritual or philosophical principles independently.

### Novel concepts participants found helpful from Buddhism

#### Buddhist mind science

Participants reported that the application of mindfulness within the framework of Buddhist mind science proved advantageous in comprehending the processes of emotions. This broader perspective allowed them to gain insights into the mechanics of emotions, enabling a clearer understanding of biases and providing a basis for effective application of appropriate antidotes. Understanding the intricate workings of the mind through mindfulness not only enhanced participants’ awareness but also empowered them to address emotional challenges with greater precision and efficacy.

Mindfulness is part of the broader Buddhist framework of how the mind and perception operate. According to the Lorig, the fundamental teachings/text that explains awareness and knowing (Tib. *blo rig*), mindfulness is one of 51 mental factors of how Buddhists understand the process of consciousness. Mindfulness is also used in understanding how the world works and seeing phenomena/reality clearly without one’s delusions and biases. There are five ‘omnipresent’ mental factors that are always present in one’s consciousness and another 46 that are utilized depending on the situation and needs (Karunananda et al., 2015). This is akin to having different personality qualities that are utilized depending on the situation. For example, fortitude is utilized when you want to quit something or maybe humor if you want to relieve tension. These qualities are always there but not ‘activated’ until needed.

Buddhism uses meditation practices to strengthen the wholesome/virtuous mental factors and reduce the unwholesome/non-virtuous ones. The West is familiar with mindfulness meditation, but traditionally, mindfulness meditation is used in tandem with concentration/calm-abiding meditation (‘shamata meditation) to improve one’s analytical ability. So, in addition to mindfulness, *concentration* is a neutral mental factor that is responsible for being able to focus and hold a mental construal in the mind (Purser and Milillo, 2015; Thanissaro and DeGraff, 2012). Being able to hold and focus on a problem in the mind allows you to better analyze its qualities and determine whether the assessment one has made is accurate and conducive in fostering a moral life. This analytical process might incorporate several other mental factors that each have their own cognitive process in the Buddhist framework (e.g., *wisdom* understands the true nature of phenomena).

**Figure 1:**
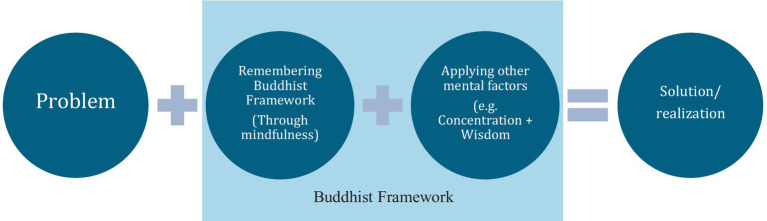
Traditional Buddhist application of mindfulness.

To illustrate how mindfulness might be applied in a Buddhist application, *aspiration* seeks or aspires to engage an object, *resolution* sets the mind on that specific object, *mindfulness* keeps the mind fixed on that object without forgetting it, *concentration* holds the mind single pointedly on that object, and *wisdom* differentiates the attributes of that object. Simply put, the problem is analyzed in the backdrop of the Buddhist framework (through the ability of *mindfulness*/remembering) and then *concentration* is applied, along other mental factors such a *wisdom*, to analyze the issue and hopefully come to some realization or solution (see Figure 1).

Concentration and the mental factors framework are typically excluded in Western iterations of mindfulness, but in traditional practices, mindfulness and concentration are often used in tandem to gain better insights into Buddhist theories and liberation alongside the other mental factors. Buddhists use mindfulness to recall past learnings, apply a framework of ethical discernment to present circumstances, and subsequently undertake the appropriate actions to enhance one’s life, worldview, and mental well-being. There is a distinction and degree of judgment for the Buddhist that is not emphasized in the West. Participants found the broader framework beneficial, extending beyond a mere conception of mindfulness to encompass a more comprehensive understanding of their perceptions and human experience. This expanded perspective facilitated a more holistic and insightful grasp of not only mindfulness but also their emotions and mental health. Integrating the mental factors into existing MBIs as a follow-on or second-phase intervention could help deepen participants’ understanding of their internal processes.

#### Dependent arising, emptiness and interconnectedness

In the survey, dependent arising was the most recommended Buddhist concept that could add value to Western MBIs, with emptiness being the third. These concepts, described below, are often indirectly taught in Western mindfulness, but in the Buddhist teaching of mindfulness, they are foundational. For example, dependent arising is a key concept of the Sattipatthana Sutra (a foundational Buddhist text on Mindfulness, mentioned above; Analayo, 2003). This Sutra examines the corporeal form and its deconstruction into constituent elements, such as organs and bodily fluids. In Western adaptations of mindfulness, such as the MBCT program, elements of the concept of emptiness are incorporated, particularly in sessions 2, 4, and 5, with an emphasis on mitigating reactivity. However, the emphasis on dependent arising, which is a hallmark of the Buddhist rendition and something participants found useful, is not as pronounced in Western iterations. These concepts are only recently emerging in the literature, as possibly separate to mindfulness interventions (Van Gordon et al., 2021).

*Dependent arising* challenges the perception of phenomena as fixed entities, and conceiving them as such leads to delusions and suffering. Phenomena are characterized by dependence such as dependence on constituent parts *or* dependence on causes and conditions. For example, viewing a cup or the human body as an unchanging, single concept is a fallacy as they are not independent, self-arisen states. They consist of interconnected parts that depend on each other to form the whole (e.g., handles and walls for a cup or extremities and organs for a body). They can also be constantly undergoing transformation (e.g., clay and cells break down constantly over time). The notion of dependent arising promotes the interdependent and causal nature of phenomena, emphasizing their reliance on various elements and their changing characteristics.

Participants mentioned emptiness is more than dependent arising but these are often linked in Buddhism. For example, a renowned Buddhist scholar Ja Tsongkhapa’s famous insights were that emptiness or ‘voidness’ means dependent arising and dependent arising means voidness (Berzin, 2024). Therefore, it is understandable that the participant’s comments reflected the integration.

*Emptiness* is a broad, complex and foundational concept in Buddhism that is difficult to summarize in a few words. For the sake of simplicity, it can be distilled as the acknowledgment that our perceptions of phenomena are often distorted, leading to inaccurate representations that leads to delusions and, eventually, suffering (Van Gordon et al., 2021; Van Gordon et al., 2017). Emptiness suggests that phenomena lack inherent or fixed meaning and are instead dependent on various factors for their existence and interpretation. For example, when one labels phenomena as inherently good or bad, we impose fixed definitions upon them, distorting one’s understanding. Like with dependent arising, fixed and biased perceptions confine phenomenon within rigid conceptual frameworks, limiting their flexibility and accuracy. This increases delusions and is a root cause of suffering, according to Buddhism. To illustrate, a table may be conventionally perceived as a surface for placing objects, but this fixed label overlooks its potential alternative functions, such as serving as a step ladder.

Emptiness teaches one to recognize the fluidity and interconnectedness of phenomena, urging us to dissipate our rigid conceptual boundaries and perceive reality more expansively. In Buddhism, this is particularly applied when contemplating ones ridged self-concept and the suffering caused from one’s ego-defense. Well-being stems from dismantling the sense of self and its attachments, as beliefs in in a fixed self, contribute to a decreased sense of connectedness and egoistical behavioral responses (Van Gordon et al., 2021).

Buddhists posit that phenomena are inherently neutral or ‘empty,’ devoid of inherent qualities. It is individuals who attribute positive or negative characteristics to phenomena, leading to suffering. Buddhist’s objective is to employ tools, like meditation, to scrutinize phenomena, allowing individuals to recognize their own biases. Through this process, the aim is to perceive all phenomena as inherently empty, devoid of fixed attributes and ultimately liberating oneself from the causes of suffering.

When participants integrated these insights with the theory of dependent arising, they recognized the contextual nature of their existence within interconnected parts, emphasizing the vital interdependence of communities for survival. The interconnectedness led to an increase in their gratitude and compassion and a decrease in their independent sense of self. This appeared to be an antidote for strong emotions that are based on a single viewpoint or perspective. While research on dependent arising and emptiness is a nascent and expanding field, current studies support participants’ experiences. For example, when advanced meditators induced a state of emptiness, they were able to improve their mystical experiences, non-attachment, compassion, and mood, compared to mindfulness (Van Gordon et al., 2019) and was a key to eliciting profound spiritual experiences (Van Gordon et al., 2018).

While these concepts are not explicitly taught in most MBIs, many programs still address the understanding that perception is shaped by subjective bias and one can be trained to become less reactive. This theme is particularly evident in programs such as MBSR and MBCT, where participants are encouraged to observe thoughts and emotions without identification or judgment.

Acknowledging this parallel could help bridge traditional and contemporary understandings but it is not easily understood by everyone (Shonin and Van Gordon, 2016; Van Gordon et al., 2021). For those who can, emptiness and dependent arising present a practical, logical, and accessible approach for the Western mind, serving as an antidote to emotional distress and contributing to a better understanding of reality. Furthermore, it underscores the connection between one’s mindful state and the well-being of others, aligning with the broader objectives of a moral life and spiritual path. Exploring these concepts for integration into current or follow on programs is an area for future research.

## Limitations

While this study aimed to *explore* the differences between traditional Buddhist and Western psychological approaches to mindfulness, certain limitations should be acknowledged. Firstly, the Buddhist objective of ending cyclic existence represents an important and fundamental difference to Western interventions. Trying to incorporate Buddhist worldviews and participants’ comments poses challenges in accurately conceptualizing and explaining these complex philosophical frameworks.

Additionally, many participants had limited prior exposure to MBIs, and for those with experience, the specific interventions they had encountered were not clearly identified. This lack of familiarity may have influenced their perspectives and responses. Furthermore, participants’ decision to attend a 30-day Buddhist meditation retreat and actively engage with Buddhist teachings may have contributed to an inherent affinity toward Buddhist principles, potentially introducing bias into their views.

Moreover, the sample predominantly comprised well-educated individuals participating in a retreat held at a Tibetan Buddhist monastery, with some responses limited in depth. As such, the findings should be seen as offering conceptual insight into potential directions for integration, rather than making claims of broad generalizability.

Finally, it is essential to recognize that MBIs are not intended to replicate Buddhist teachings fundamentally, suggesting that the interpretations and applications of Buddhist concepts within Western psychological interventions may inherently deviate from their traditional forms. Additionally, comparing Buddhism’s comprehensive worldview to an intentionally secularized intervention might not be fair. Mindfulness programs are designed to operate within pluralistic clinical environments, where embedding a fixed worldview may conflict with the intention to remain philosophically neutral and accessible to diverse populations.

## Conclusion

In conclusion, this study affirms that while Western approaches have successfully assimilated several insights from participants’ suggestions, there exists potential to embrace further wisdom from traditional Buddhist practices. Participants highlighted the importance of extending mindfulness conceptualization in the West and advocated for a more expansive Buddhist worldview that transcends conventional mindfulness and emotional reactivity so that people can gain greater benefits from their practice. Noteworthy examples of these broader perspectives include the intricate Buddhist mind science, offering a comprehensive framework for perception and cognition, as well as the concepts of emptiness and dependent arising. The value of emptiness is already beginning to be examined in a Western context. Future research could explore integrating some of these Buddhist perspectives into Western psychological interventions, with a specific emphasis on advancing mindfulness practices for a more holistic approach to mental well-being.

## Data Availability

In compliance with the requirements of the Monash University Research Ethics Committee, the datasets presented in this article are not readily available because participants did not consent to sharing their data. Requests to access the datasets should be directed to Dr Frances Shawyer at frances.shawyer@monash.edu.
