# Supplementary material for: Mindfulness-based interventions: what more can the West learn from Buddhism? A fieldwork study
Source: Front Psychol. 2025 Jun 23;16:1579575. doi: 10.3389/fpsyg.2025.1579575 (PMC12230017; doi:10.3389/fpsyg.2025.1579575)
Supplement: Supplementary file 1 [file Data_Sheet_1.docx]

**Appendix A**

**Demographic, Buddhist and Meditation Questions (Survey)**

| Demographic Questions | | Answer options | | | Follow on questions |
| --- | --- | --- | --- | --- | --- |
| 1. Which age group do you fit into? | | - Less than 20 years - 20s - 30s - 40s - 50s - 60+ years - Prefer not to say | | |  |
| 2. What is your gender? | | - Female - Male - Another term - please supply __________________ - Prefer not to say | | |  |
| 3. Where were you born? | |  | | |  |
| 4. What is the main language spoken at home? | |  | | |  |
| 5. What is your highest level of education? | | - Primary school - Secondary school - Diploma (Inc Associate Diploma) - Technical qualification - Bachelor Degree - Postgraduate qualification(s)   ____________________________ | | |  |
| Background questions related to Buddhism and spiritual practice | |  | | |  |
| 6. For how many years would you say you have taken an active interest in Buddhism? | | Less than 1, 2, 3, 4, 5+, 10+ | |  |  |
| 7. Roughly how many days in total have spent in a retreat setting other than the Kopan course? | |  | |  |  |
| 8. What is your main spiritual background or religion? | | - Non-religious - Spiritual - Buddhism - Christian - Hinduism - Islamic - Judaism - New Age - Other: _________________ | |  |  |
| 9. Do you consider yourself a Buddhist? | | - Yes - No | |  |  |
| Background questions related to meditation practice and mindfulness training | | |  |  |  |
| 10. How often did you meditate prior to the November course? | | | - Multiple times a day - Daily - Multiple times a week - Weekly - Monthly - Only at retreats - Never |  |  |
| 11. What type of meditation do you do most often? | | |  |  |  |
| 12. Have you completed a mindfulness-based intervention before (as based in Western psychology)? | | | - Yes - No | If yes, please specify: |  |
| Core research questions | | |  |  |  |
| 13. What Buddhist-based concepts, teachings or practices have had the most positive impact on your life? | | | Open |  |  |
| 14. What Buddhist-based concepts or practices have you found most challenging or in tension with your pre-existing culture and traditions? | | | Open |  |  |
| 15. What Buddhist concepts and/or practices do you think would have helped or enhanced the mindfulness-based intervention you completed? | | | I have not completed a mindfulness-based intervention  Open |  |  |
| 16. What Buddhist concepts and/or practices do you think would be valuable to add to Western Mindfulness-Based Interventions? | | | Hell realms, mindfulness, karma, rebirth, emptiness, dependent arising, four Noble truths, attachment and aversion, bodhicitta, Boddhisatva, Other | Why? (Option and open) |  |
| 17. From your experience of Kopan and Buddhism more generally what do you think the West needs to learn in relation to meditation or being mindful? | | | Meditation: Open | Mindfulness: Open |  |
