# Supplementary material for: Mindfulness-based interventions: what more can the West learn from Buddhism? A fieldwork study
Source: Front Psychol. 2025 Jun 23;16:1579575. doi: 10.3389/fpsyg.2025.1579575 (PMC12230017; doi:10.3389/fpsyg.2025.1579575)
Supplement: Supplementary file 2 [file Data_Sheet_2.docx]

**Appendix B**

**Semi-Structured Interview Questions**

**Demographics questions:**

- What is your age?
- What is your gender?
- Where were you born?
- What is your highest level of education?

**General/intro questions:**

- - Could you please tell me a bit about your own spiritual/religious background and culture?
  - Can you say a few things about your experience of the Kopan Monastery Lim Rim Meditation course?
  - What did you learn from the course that you brought home with you?
  - What, if anything, did you find were unpleasant aspects of the course?
  - What did you most enjoy - what was the most fun?
  - Can you say a few things about your experience with the meditations during the course?
    - Which ones did you find useful or helpful?
    - Which ones might you continue at home?

**Mindfulness-related questions:**

- - What is your understanding of mindfulness?
  - What experience have you had with Mindfulness as practiced in the West before attending this course?
    - [if they **have** previouse MBI experience]
      - Do you think there are any differences between the mindfulness you have learnt in the West to the mindfulness taught at the Kopan course or Buddhism more generally?
      - If yes,
        - What are the differences?
        - Why do you think there are differences?
        - What do you think are the advantages and disadvantages of the differences?
      - What key points about mindfulness do you think should be developed in the West?
    - [if they **not** have previouse MBI experience]
      - What was easy to understand about mindfulness?
      - What was difficult to understand?
      - What key points about mindfulness do you think should be developed in the West?

**Integrating the West and Buddhism:**

- - What are the hardest parts of the course or Buddhism that you have learnt about?
    - What helped or what would you need to make it easier?
  - What teachings from the course did you find challenging or in tension with your pre-existing culture and traditions?
  - What teachings from the course did you find easy to integrate with your pre-existing culture and traditions?
  - Overall, what aspects of the course do you think could be usefully integrated into Western mindfulness-based interventions to reduce suffering?
  - Beyond mindfulness, what key teachings or practices from Buddhism do you think could be usefully integrated into Western psychological interventions?
